# Chorea, a little-known manifestation in systemic lupus erythematosus: short literature review and four case reports

**DOI:** 10.1186/1546-0096-11-36

**Published:** 2013-10-16

**Authors:** Sofia Torreggiani, Marta Torcoletti, Federica Cuoco, Giancarla Di Landro, Antonella Petaccia, Fabrizia Corona

**Affiliations:** 1 Pediatric Rheumatology, Fondazione IRCCS Ca' Granda Ospedale Maggiore Policlinico, Via Commenda 9, Milan 20122, Italy

## Abstract

Chorea is a movement disorder that may be found in children due to several causes. Here we focus especially on Systemic Lupus Erythematosus associated chorea. First we outline its epidemiology, hypothesized pathogenesis, clinical presentation and treatment, then we report four significant clinical cases, which represent well the extreme variability of set of symptoms that may accompany lupus chorea. Our experience, according to literature, suggests that choreic movements in a child should alert the pediatrician and lead him to investigate a potential neurological involvement of Systemic Lupus Erythematosus.

## Background

The term chorea (choreia = dance) defines a movement disorder and a syndrome characterized by not repetitive sudden involuntary movements, that may involve any portion of the body.

Chorea may occur in the context of various diseases (Table 1). Even if the most common etiology of chorea in pediatric patients is the autoimmune form of post-streptococcal origin [1], chorea may be also a complication of Systemic Lupus Erythematosus (SLE).

**Table 1:** Differential diagnosis of chorea in pediatric age

**Etiology**	**Specific conditions**
Central nervous system infections	Herpes simplex virus, varicella zoster virus, paramyxovirus (measles), HIV, lyme disease, mycoplasma
Autoimmune	Sydenham chorea, systemic lupus erythematosus, antiphospholipid syndrome, Neuro-Behcet, primary central nervous system vasculitis
Neurodegenerative, metabolic, genetic	Huntington’s disease, Phenylketonuria, Wilson disease, mitochondrial pathologies
Vascular	Stroke, post-cardiac transplant, arteriovenous malformation, moya-moya disease
Endocrinological	Hyperthyroidism
Toxic	Anticholinergic toxicity, dopamine antagonists, carbon monoxide, neuroleptic drugs.

Modified from Luca NJ et al. *Paediatr Child Health* 2011 [2].

SLE is a systemic autoimmune disease characterized by the presence of autoantibodies and multiorgan involvement. Clinical presentation of the disease is extremely variable. The classification of SLE is established on the basis of specific criteria [3], which include only psychosis and seizures as nervous system manifestations; nevertheless also other neuropsychiatric disorders are associated with SLE (neuropsychiatric SLE). Neuropsychiatric SLE is reported in 20% to 95% patients with pediatric SLE [4]. In 1999 the American College of Rheumatology (ACR) compiled a standardized nomenclature, providing case definitions for 19 neuropsychiatric syndromes seen in SLE as described in Table 2[5].

**Table 2:** Neuropsychiatric syndromes observed in SLE

**Central nervous system**	**Peripheral nervous system**
● Aseptic meningitis	● Acute inflammatory demyelinating polyradiculoneuropathy (Guillain-Barré syndrome)
● Cerebrovascular disease	
● Demyelinating syndrome	
● Headache (including migraine and benign intracranial hypertension)	● Autonomic disorder
● Movement disorder (**chorea**)	● Mononeuropathy, single/multiplex
● Myelopathy	● Myasthenia gravis
● Seizure disorders	● Neuropathy, cranial
● Acute confusional state	● Plexopathy
● Anxiety disorder	
	● Polyneuropathy
● Cognitive dysfunction	
● Mood disorder	
● Psychosis	

The American College of Rheumatology nomenclature and case definitions for neuropsychiatric lupus syndromes. *Arthritis Rheum* 1999, 42(4):599–608.

Among movement disorders described during SLE, chorea plays a leading role, being the most frequent one (0-5% of all patients with pediatric SLE) [4].

The pathogenesis of lupus-associated chorea is still unclear, but it may be related to a vascular, neuronal and glial injury. Vascular damage may be inflammatory and/or thrombotic. Ischemic pathology is often due to autoantibodies, mainly antiphospholipid antibodies (aPL).

Antiphospholipid antibodies seem to play a decisive role in neuropsychiatric SLE pathogenesis. Positive finding of aPL can be based on an abnormal serum level of IgG or IgM anticardiolipin antibodies (aCL), on an abnormal serum level of IgG or IgM anti-beta 2 glycoprotein I antibodies or on a positive test result for lupus anticoagulant (LAC) using a standard method. The prevalence of anti-beta 2 glycoprotein I antibodies is higher in SLE patients with neuropsychiatric disease compared with those without. Antiphospholipid antibodies are more frequently identified in patient with lupus chorea, than in all patients with SLE [6]. It was demonstrated an association between a persistently positive LAC and chorea [7].

In some studies it was emphasized the direct role of aPL and anti-beta 2 glycoprotein I antibodies, which would act not only through their procoagulant effects, but also causing direct damage to neuronal cells. It was shown that IgG isolated from the serum of patients with aPL at high titer are able to depolarize synaptoneurosomes purified from cerebral cortex of rats: this was the first evidence of direct neuronal damage, that could explain neuropsychiatric manifestations in the absence of documentable thromboembolic damage in the CNS and other abnormalities at MRI [8].

In the pathogenesis of diffuse neuropsychiatric symptoms, also antineuronal antibodies, antibodies against ribosomal P-protein and cytokines have been documented to play a role [9].

Some data suggest that also sex hormones may be involved in the pathogenesis of chorea, especially after case reports of SLE chorea during pregnancy (Chorea Gravidarum) [10].

Chorea can appear at any time during pediatric SLE, during both the active disease and the clinical remission. It can represent the first clinical manifestation of SLE [11], preceding the other signs that are among the classification criteria [12]; alternatively, it can appear at a later time, even after several months from the onset of the disease. Chorea typically manifests with jerky, involuntary and purposeless movements, mainly involving the four limbs. Patients with chorea may have difficulty walking and keeping the upright posture; loss of coordination of tongue and hands muscles can cause dysarthria and difficulties in writing. All symptoms are accentuated by stress or excitement, while they are reduced during rest and sleep.

Difficulties in the diagnosis of neuropsychiatric SLE lie mainly in the fact that clinical symptoms, often relevant, may occur without any detectable lesion at neuroimaging [13]; conversely anatomical and/or functional alterations are described in patients without symptoms or signs of neuropsychiatric pathology [14]. Magnetic resonance imaging (MRI) is more sensitive than computed tomography (CT) for neuropsychiatric SLE-associated lesions, even if neuroimaging findings don’t always relate specifically with the clinical picture. Electroencephalographic (EEG) evaluation presents the same problem.

Positron emission tomography (PET) scan using [^18^ F]deoxy-glucose has shown hypermetabolism in the contralateral striatum in patients with SLE chorea compared with normal controls [15]. Similar findings were also observed in other forms of suspected autoimmune chorea [16], in contrast to the striatal hypometabolism observed in vascular and hereditary neurodegenerative chorea [17].

Quantitative imaging techniques such as volumetric magnetisation transfer imaging (MTI) are able to show diffuse tissue abnormalities not visible on normal MR analysis. MTI histogram peak height values in neuropsychiatric SLE were lower than in controls or patients with SLE without neuropsychiatric symptoms. Moreover, MTI parameters in the active phase of neuropsychiatric SLE are different from those in the chronic phase [18].

The inadequate knowledge of pathogenetic mechanisms and the lack of randomized-controlled trials imply that the treatment of chorea in SLE is not yet completely codified. The most recent EULAR recommendations for the management of SLE with neuropsychiatric manifestations advise to treat patients with persistent choreic symptoms with dopamine antagonists; it is also recommended to consider antiplatelet therapy in aPL positive patients. Anticoagulation should be reserved for patients with positive aPL who experienced a clinical thromboembolic event [19]. Glucocorticoids in combination with immunosuppressive agents (azathioprine, cyclophosphamide) may be used to control NPSLE disease activity [20].

## Case presentation

83 patients diagnosed with SLE (13 males and 70 females) are followed at our division of Pediatric Rheumatology. At the onset of SLE symptoms, their average age was of 11,3 +/− 2,7 years (range 1–16 years). 43 patients had neuropsychiatric involvement; among them 5 presented chorea (11,6% of the patients with neuropsychiatric SLE and 6% of the patients with SLE). In our clinical records, in agreement with the literature, we have found a correlation between chorea and aPL positivity. Patients with chorea showed a more severe disease and a worse prognosis.

### Case 1: chorea, arthritis, SLE

A 12-year old previously healthy girl was admitted to our division due to ataxic gait and choreic movements. She also complained of asthenia, difficulty concentrating, depression and decline in school performance. Blood tests performed after admission showed: positive ANA (1:2560), aCL, anti-DNA antibodies and Coombs test; LAC was negative. The EEG documented a modest alteration of the overall organization, expressed in a symmetrical manner in the two hemispheres, and independent focal slow-wave abnormalities in the left temporal and right occipital, where sporadic epileptiform abnormalities were also found. MRI documented nonspecific punctate hyperintensities bilaterally at the level of the medial fronto-parietal subcortical white matter. During hospitalization, the patient developed arthritis that involved all the fingers of the hands, wrists and knees; choreic movements of the upper limbs were also present. SLE diagnosis was confirmed by the presence of the classification criteria and supported also by the neurological involvement (chorea). Intravenous therapy with steroids (methylprednisolone acetate, 20 mg/ kg/day administred for 3 consecutive days) led quickly to the complete remission of the choreic symptoms. Treatment was prosecuted with oral prednisone (1 mg/kg/day), with gradual tapering thereafter. During the subsequent 5 years of follow-up, the patient no longer experienced movement disorders. On the other hand, at the age of 13, she presented a major renal involvement (proliferative glomerulonephritis, Class III), hypertension, deep vein thrombosis.

### Case 2: epilepsy, coma, vascular malformation, chorea, SLE

The girl, carrier of glucose-6-phosphate dehydrogenase deficiency, enjoyed good health until the age of 3, when she had seizures characterized by loss of consciousness with hypotonia. She was diagnosed with idiopathic partial epilepsy and was treated first with carbamazepine and then with valproic acid, with no more evidence of seizures, so that antiepileptic therapy was suspended.

At the age of 6, she had an important episode of headache with vomiting and backache, followed by a progressive state of coma. She was admitted to the local hospital. Brain CT scan documented a massive left frontal intraparenchymal hematoma with endoventricular haemorrhage. The girl started therapy with steroids and tranexamic acid, with marked improvement of the clinical picture. Subsequently, CT and MRI described resorption of the hematoma, but showed in the same location a porencephalic cavity communicating with the frontal horn of the lateral ventricle. Angiography documented the presence of an arteriovenous vascular malformation, subsequently removed by surgery. The exams showed positive LAC, increased APTT and reduction of coagulation factors VIII and IX.

At the age of 11, the girl presented hand swellings. Blood tests showed positive ANA (1:160), positive anti-DNA antibodies, low C4 (3 mg/dL). Capillaroscopy documented nonspecific microangiopathic alterations with features of acrocyanosis. Due to suspected SLE, she was treated with steroid (deflazacort 1,2 mg/kg/day) and acetylsalicylic acid (ASA, 100 mg/day).

She experienced clinical well-being until the age of 12, when, after reduction of steroid therapy (deflazacort 0,3 mg/kg/day), swellings reappeared, involving wrists, fingers and elbows. Methotrexate therapy was started (10 mg/m^2^ once a week), with benefit; it was suspended one year later due to an increase in liver enzymes. In that occasion, at the age of 13, the girl repeated blood tests which showed: positive aCL, LAC and IgM anti-beta 2 glycoprotein I, erythrocyte sedimentation rate (ESR) 22 mm/hr, C3 44 mg/dL, C4 2 mg/dL, positive ANA (1:320), positive anti-DNA antibodies, thrombocytopenia (67000/mm^3^). Considering SLE diagnosis, azathioprine (2,12 mg/kg/day) was added to the steroid therapy (prednisone 0,5 mg/kg/day). Due to glucose-6-phosphate dehydrogenase deficiency, treatment with hydroxychloroquine was not prescribed. Two months later, the patient showed hyperkinesis in the right hemibody and especially in the tongue, with subsequent dysarthria. In the following weeks, she developed generalized chorea, mainly in the right hemibody. The MRI didn’t show changes from the previous ones. At the same time butterfly rash appeared. The girl was transferred to our division, where diagnosis of SLE was confirmed, since during the clinical history of the patient the classification criteria were present. Chorea was attributable to neuropsychiatric lupus. Choreic symptoms regressed after administration of intravenous methylprednisolone acetate (20 mg/ kg/day for 3 consecutive days), therapy was prosecuted with oral prednisone (0,75 mg/kg/day). Azathioprine therapy was continued with the same dose; ASA was suspended.

**Figure 1:**
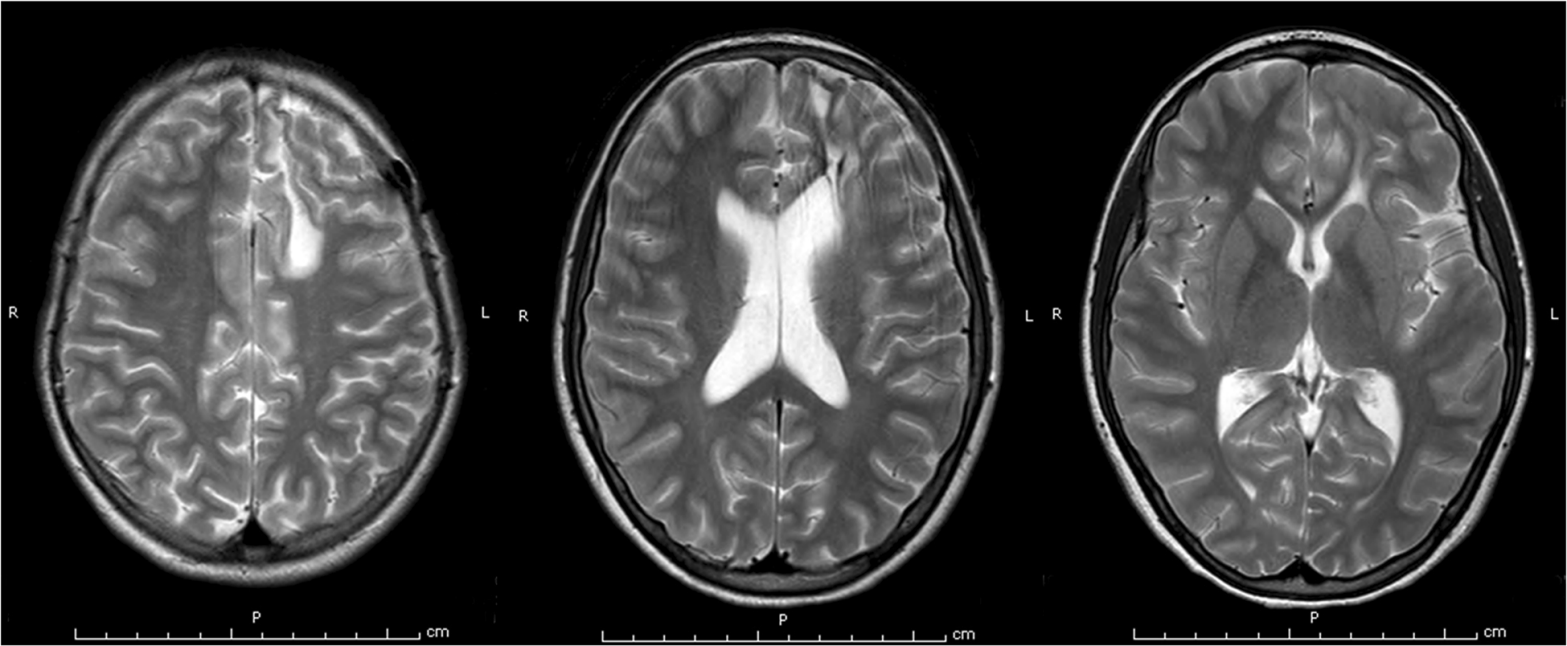
**Brain MRI of case 2.** T2-weighted images on brain MRI showing, in the left supratentorial region, the known surgical outcomes (with left frontal craniotomy) and a malacic area, communicating with the omolateral frontal horn. No further significant signal alterations are evident.

At the age of 14 years, following the tapering of corticosteroid therapy, choreic movements reappeared. As in the whole clinical history, also in this occasion blood tests showed positive LAC. The MRI didn’t reveal recent parenchymal lesions (Figure 1). Treatment with intravenous methylprednisolone acetate (20 mg/kg/day, administered for 3 consecutive days) rapidly improved the symptoms; therapy was prosecuted with oral prednisone (0,75 mg/kg/day). In the 10 months of follow-up the girl didn’t show other choreic movements.

### Case 3: fever, myalgias, aphtae, opisthotonus, chorea, diffuse proliferative glomerulonephritis, SLE

The patient was in good health until the age of 14, when she presented hyperpyrexia, diffuse myalgias, oral aphtae, an episode of marked agitation, opisthotonus and diffuse hyperalgia.

She underwent an EEG, which showed widespread brain injury; brain CT scan documented in the left hemisphere a faint subcortical fronto-temporal hypodensity, with blurred limits. Antibiotic, antiviral and anti-edema therapy was started, but 3 days after admission, choreic movements and incomprehensible speech appeared. Blood tests showed increased inflammatory markers (ESR 110 mm/hr, C-Reactive Protein 50 mg/dL - normal values <1 mg/dL), hypocomplementemia, positive ANA and anti-DNA, negative LAC, microscopic hematuria and proteinuria; aCL were not investigated, since at that time it was not yet a routine test. One week after the onset of symptoms, the patient was transferred to our division: she still had choreic movements of the four limbs; her gait was ataxic and she was not able to walk with eyes closed. The presence of oral ulcers, pathological proteinuria, positive ANA and anti-DNA allowed a diagnosis of SLE. She started steroid therapy with intravenous methylprednisolone acetate (20 mg/ kg/day), administered for 3 consecutive days, prosecuted with oral prednisone (1 mg/kg/day). In the following days, choreic symptoms worsened: brain MRI showed punctate subcortical hyperintensities in both cerebral hemispheres, suggestive of a vasculitic process. Failure of steroid therapy, neurological symptoms and important renal involvement (diffuse proliferative glomerulonephritis, Class IV) justified the use of intravenous cyclophosphamide (0,5 mg/m^2^ of body surface area, once a month for 6 months and then once every two months for other 6 months). 15 days after the first cyclophsphamide pulse, the choreic symptoms resolved, also with the use of haloperidol. In the last 8 years of follow-up, being the girl treated with low-dose steroid therapy, values of proteinuria normalized and the patient no longer showed choreic symptoms or other neurologic manifestations.

### Case 4: SLE, chorea, diffuse proliferative glomerulonephritis

The child enjoyed good health until he was 10, when he started presenting asthenia, arthralgias and abdominal pain. He was admitted to another hospital, where blood tests showed microscopic hematuria, hypocomplementemia (C3 11 mg/dL and C4 3 mg/dL), positivity for ANA (1:2560), anti-DNA and LAC. In suspected SLE, the child was sent to our division, where he showed the butterfly rash and arthritis involving both wrists, all proximal interphalangeal joints and metacarpophalangeal joints of the second and third finger of both hands. In view of the clinical examination, the renal involvement, the positivity for ANA, anti-DNA and LAC, diagnosis of SLE was confirmed, also supported by hypocomplementemia and pathological capillaroscopic findings. Intravenous methylprednisolone acetate (20 mg/kg/day) was administered for 3 consecutive days, followed by oral prednisone (1 mg/kg/day, with gradual tapering thereafter).

Seven months after the onset of the disease, when prednisone was at the dose of 0,65 mg/kg/day, choreic movements of the four limbs appeared; butterfly rash and oral apthae were also present. He was therefore hospitalized a second time; brain MRI showed multiple lesions of vasculitic origin in both hemispheres, while EEG results were normal. After the detection of proteinuria (1,6-2 g/24 h), renal biopsy was performed and documented diffuse proliferative glomerulonephritis, Class IV. Blood tests showed positive LAC and aCL and severe thrombocytopenia (3000/mm^3^), which required a platelet transfusion. Intravenous methylprednisolone acetate (20 mg/ kg/day) was administered for 3 consecutive days; tiapride hydrochloride was used as a symptomatic therapy; oral treatment with prednisone (1 mg/kg/day) followed. Due to persistent thrombocytopenia, intravenous immunoglobulin was administered for 5 consecutive days, with progressive increase in the number of platelets. Severe disease activity and renal involvement justified the use of cyclophosphamide (0,5 mg/m^2^ of body surface area, once a month for 6 months and then once every 2 months for other 10 months).

At the age of 13 years, only one week after the last cyclophosphamide pulse and following a reduction of steroid therapy (0,25 mg/Kg/day), left hemichorea appeared. The boy was admitted to our division; the MRI didn’t show changes from the previous one; LAC was positive (aCL were not measured). Due to mild neurologic symptoms, treatment with tiapride hydrochloride was not considered necessary. Intravenous methylprednisolone acetate (20 mg/ kg/day) was administered for 3 consecutive days, with improvement of the symptoms; tapered oral therapy with prednisone followed. During 9 years of follow-up after the last choreic manifestations, the boy didn’t experience other neurologic symptoms, but at the age of 18 worsening of the renal disease (switch from Class IV to Classes II and V), required treatment with mycophenolate mofetil.

## Discussion

Neuropsychiatric SLE was found in 51,8% of our SLE patients; this prevalence is included in the wide range reported in literature (20-95%). On the contrary, prevalence of chorea was slightly higher: we recorded it in 6% of our SLE patients (instead of 0-5% [4]).

Clinical features of SLE associated chorea in our patients are summarized in Table 3.

**Table 3:** Clinical features and outcomes of SLE associated chorea in our patients

**Case**	**1**	**2**	**3**	**4**
Onset of chorea	12 years	13 years	14 years	11 years
	At the onset of SLE	2 years after SLE onset	At the onset of SLE	7 months after SLE onset
Positive LAC and/or aCL	Yes	Yes	-	Yes
Neuroimaging	Not specific lesions	Picture mainly characterized by the porencephalic cavity and the previous surgery	Vasculitic findings	Vasculitic findings
Efficacy of high-dose steroid therapy	Yes	Yes	No	Yes
Recurrence of chorea	No	1 year after the first episode	No	2 years after the first episode
Renal involvement	Class III	No	Class IV	Class IV and later Classes II and V

In our clinical records, chorea occurred at different times during SLE disease. In according to literature, in 3 cases it appeared relatively early [11,12]: chorea was present at the onset of SLE in two cases (1 and 3) and it appeared 7 months after SLE diagnosis in case 4. Only in case 2 chorea appeared 2 years after SLE onset. Age at the onset of chorea ranged between 11 and 14 years.

When investigated, LAC and/or aCL were positive (case 1,2, and 4); in case 3 LAC was negative, while, due to the techniques available at that time, aCL were not measured at chorea onset, before starting therapy with steroid and cyclophosphamide.

Neuroimaging showed vasculitic findings in case 3 and 4; in case 1 the lesions detected were not specific. In case 2 the picture was mainly characterized by the porencephalic cavity and the previous surgery; as in a previous case report, in which the child had experienced a cerebral infarction before developing involuntary movements [21], MRI performed at the onset of chorea revealed no new lesions.

The multiorgan involvement associated with chorea in our patients justified steroid treatment. High-dose steroid therapy was able to reduce choreic manifestations, except in case 3 in which cyclophsphamide seemed to improve the neurological picture. In order to control choreic symptoms, haloperidol and tiapride hydrochloride were also used in association with the steroid therapy, with benefit.

In cases 2 and 4 chorea recurred a second time (in case 2 after one year, in case 4 after 2 years), following a decrease of oral steroid therapy. In case 4, cyclophosphamide administered for the renal involvement did not prevent the recurrence of chorea, which appeared only one week after the last pulse; nevertheless, symptoms were milder compared to the first episode, therefore cyclophosphamide may have played a role in reducing the severity of neurologic manifestations.

All patients with chorea showed a severe SLE disease: 3 of them had renal involvement (1, 3 e 4), with different histopathological patterns (Classes II, III, IV and V). Renal disease was associated with chorea also in another case reported in the literature, even if in that patient lupus nephritis occurred before chorea [21].

Chronic and end-stage renal disease is known to be a significant cause of morbidity and mortality in SLE patients [22]; this is true also in childhood-onset SLE, where incidence of nephritis can be higher than in adult-onset disease [23]. Advanced renal scarring with significant loss of functional nephrons is not reversible, even with immunosuppressive therapy, and leads to severe consequences. The fact that chorea appears early in the disease, makes its recognition as a SLE manifestation even more important. If SLE is diagnosed, a proper therapy can be started and, even if kidney disease cannot be prevented, more severe renal complications could be avoided, improving dramatically the prognosis of the patient.

## Conclusions

It’s important to remember the association between chorea and SLE. If a patient shows choreic movements, after excluding the most frequent etiologies, it should be considered that chorea may represent the neurologic involvement of SLE. The most common pediatric chorea is Sydenham chorea, but it should be considered that its incidence is expected to decrease, due to the reduced incidence of rheumatic fever in the Western countries [24]; in Latin America, Africa, and parts of Asia, the frequency of rheumatic fever and Sydenham chorea is higher, but it shows a trend towards reduction also in these regions [25]. Lupus associated chorea, even if less frequent, deserves a special attention for its clinical relevance: if Sydenham chorea is unrecognized, the prognosis quoad vitam is not significantly affected, while SLE chorea is related to severe SLE disease, for which an appropriate treatment is essential.

Currently, the ACR classification criteria for SLE include only psychosis and seizures as nervous system manifestations, but there are valid reasons for adding to these neurological disorders also chorea: the frequency of chorea in pediatric SLE is significant; furthermore, lupus chorea usually appears early in the disease, therefore recognizing it among the criteria could be an opportunity to anticipate SLE diagnosis and offer the patients a timely therapy, potentially able to reduce systemic complications of SLE.

## Consent

Written informed consent was obtained from the patients for publication of these Case Reports and any accompanying images. A copy of the written consents is available for review by the Editor-in-Chief of this journal.

## Competing interests

The authors declare that they have no competing interests.

## Authors’ contributions

ST collected and reviewed patient data and drafted the manuscript. MT contributed to the interpretation of patient data and helped draft the manuscript. FCuoco collected and reviewed patient data and helped draft the manuscript. GDL participated in data acquisition and care of the patients. AP contributed to the conception and design of the study and helped draft the manuscript. FCorona conceived of the study and designed and coordinated the study and helped draft the manuscript. All authors critically revised the manuscript and read and approved the final manuscript.
